# Supplementary material for: Total RNA sequencing reveals gene expression and microbial alterations shared by oral pre-malignant lesions and cancer
Source: Hum Genomics. 2023 Aug 4;17:72. doi: 10.1186/s40246-023-00519-y (PMC10403884; doi:10.1186/s40246-023-00519-y)
Supplement: Supplementary file 1 — Additional file 1: Figure S1: Total number of markers per pairwise analysis. Total number of differentially expressed genes in the pairwise analysis with logFC ± 1.5 and q-val ≤ {0.01, 0.05, 0.1}. Figure S2: Enrichment of cancer-associated fibroblasts. A. Gene expression of PDGFB1, COL1A1, COL1A2, COL3A1. B. Top 50 genes from fibroblast signatures shows enrichment in PML and OSCC groups. P-values are obtained by ANOVA test. C. Association of p-EMT and fibroblasts GSVA scores. Figure S3: Enrichment of lung bronchus modules. GSVA enrichment scores from modules 5, 8, and 9 from lung bronchus premalignant lesion study in our oral PML series. P-values are obtained by ANOVA test. Figure S4: Cell-type deconvolution scores of immune sub-types. A. innate, B. adaptive types. P-values are obtained by ANOVA test. C. heatmap of abundances stratified by histopathology along with smoking and progression statuses. Figure S5: Microbial diversity analysis. A. Relative abundance of microbial genera across groups with the genus of Fusobacterium in green and Streptococcus in orange. B. Alpha diversity stratified by histopathological groups, p-value obtained by Kruskal–Wallis test. C. Beta diversity stratified by histopathological groups, p-value obtained by PERMANOVA test. D. Relative abundance in DESeq2-normalized and log2-transformed counts of the top three differentially abundant species in any of the comparisons of HkNR, Dysplasia, and OSCC with control. Q-values obtained by DESeq2-based analysis. [file 40246_2023_519_MOESM1_ESM.docx]

***SUPPLEMENTARY Figures:***

#
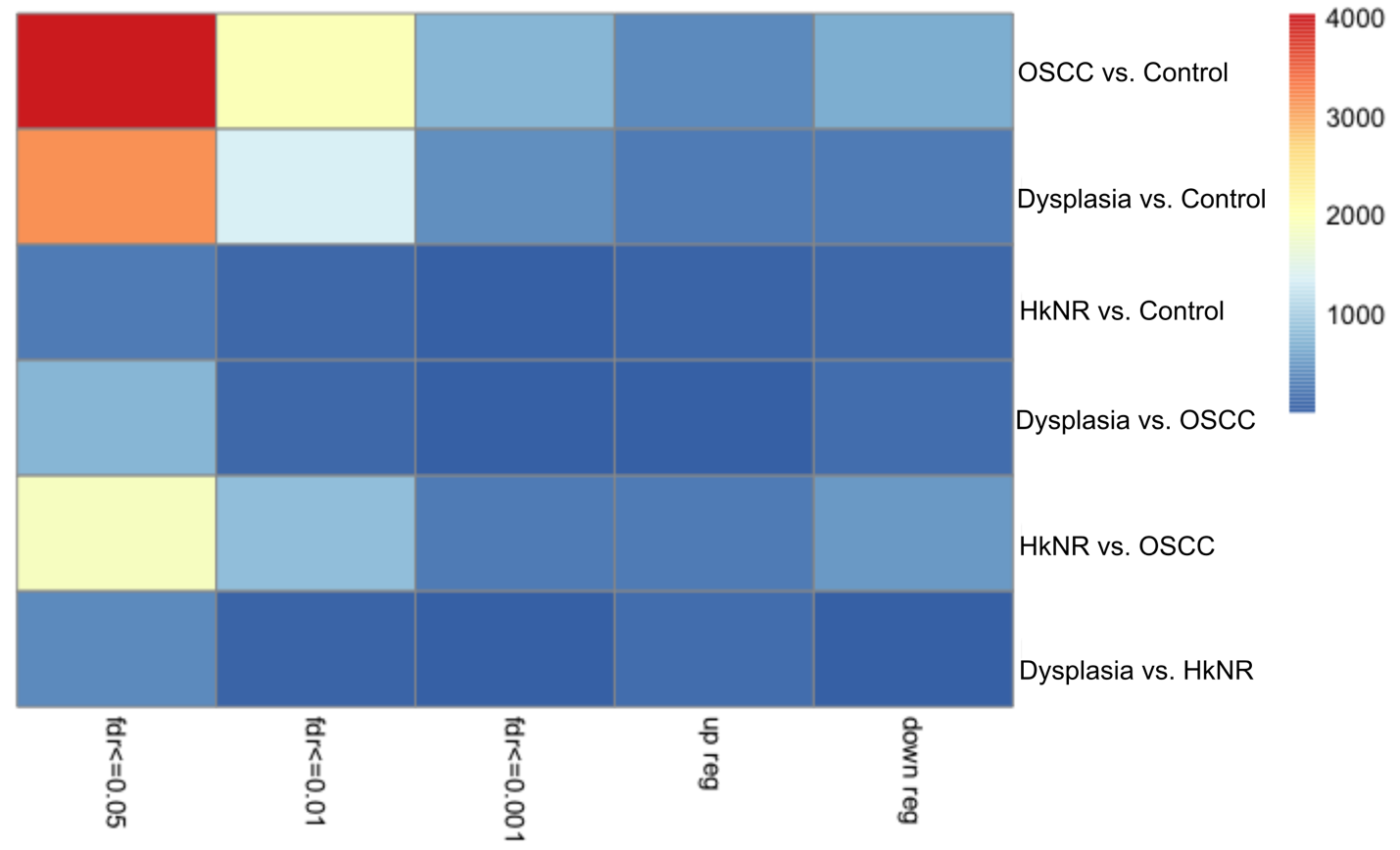


**Figure S1**: Total number of markers per pairwise analysis. Total number of differentially expressed genes in the pairwise analysis with logFC ± 1.5 and q-val ≤ {0.01, 0.05, 0.1}


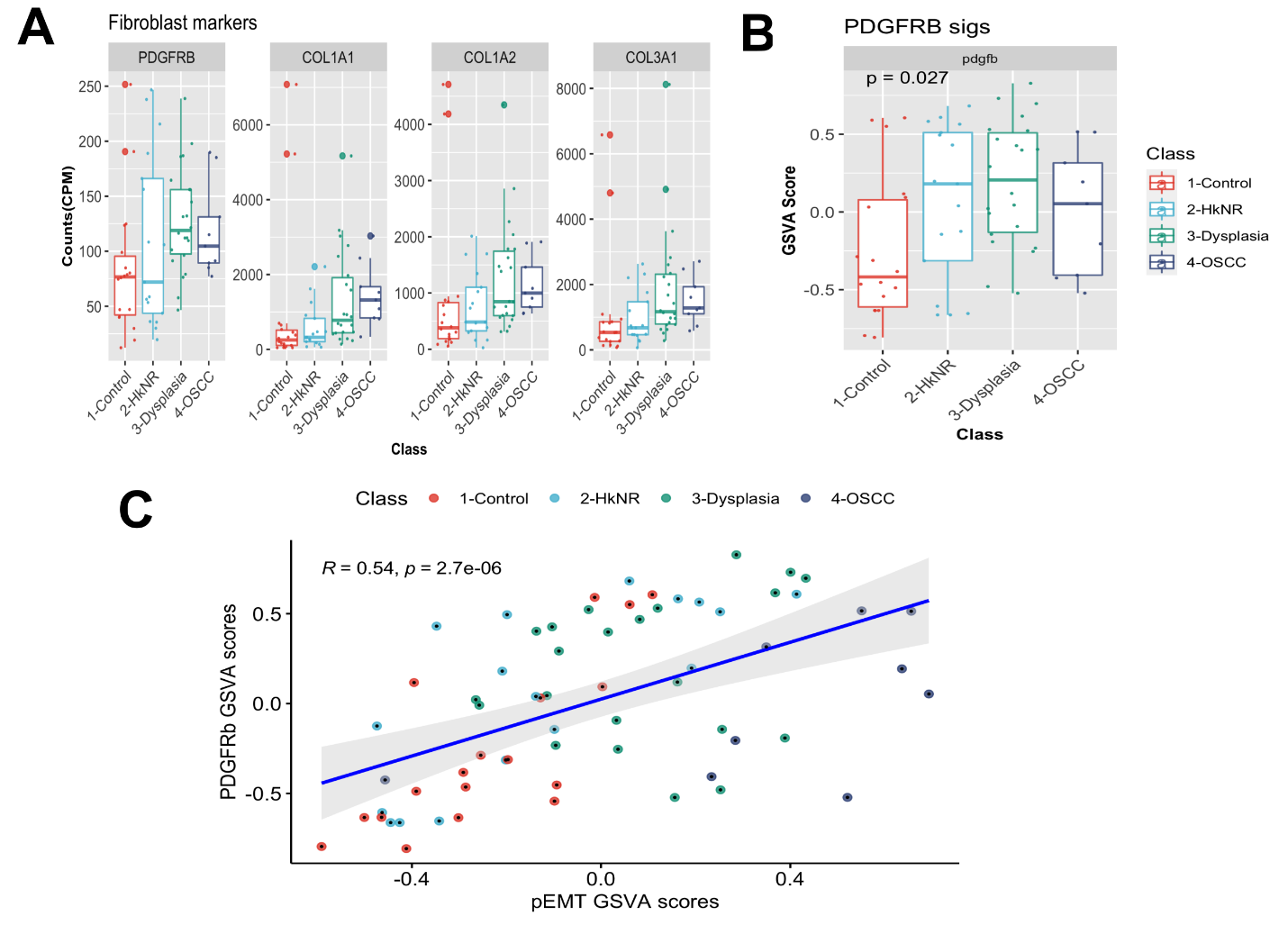


**Figure S2: Enrichment of cancer-associated fibroblasts**. **A**. Gene expression of PDGFB1, COL1A1, COL1A2, COL3A1. **B**. Top 50 genes from fibroblast signatures shows enrichment in PML and OSCC groups. P-values are obtained by ANOVA test. **C**. Association of p-EMT and fibroblasts GSVA scores.


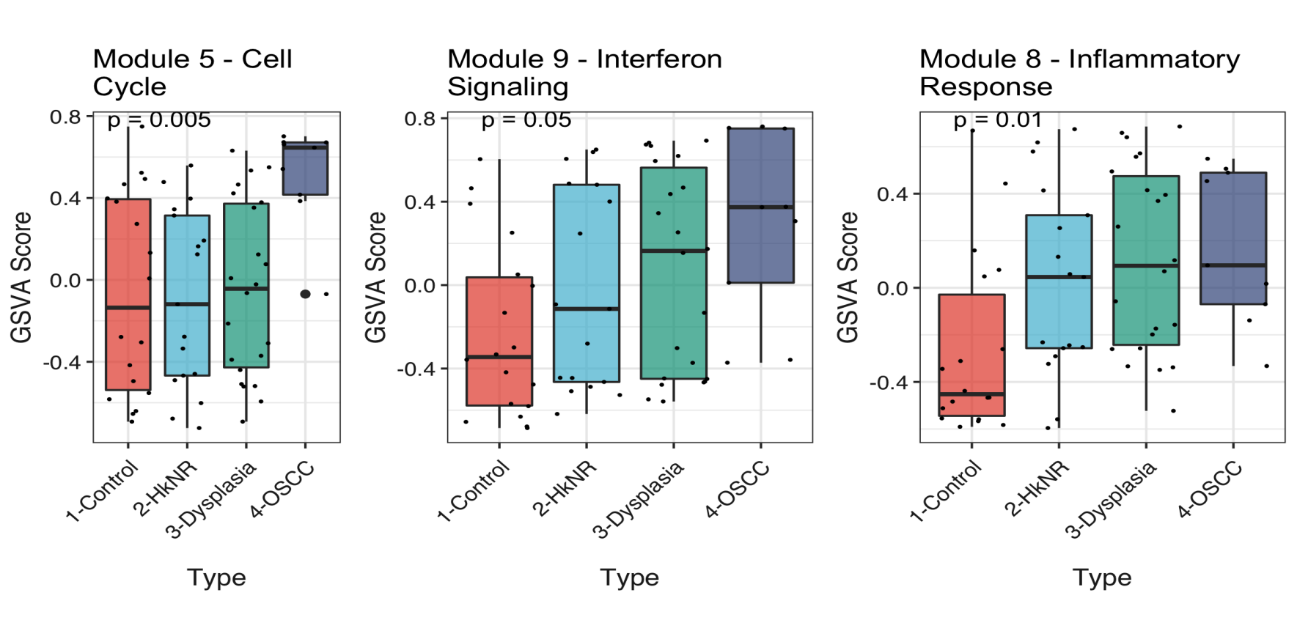


**Figure S3: Enrichment of lung bronchus modules**. GSVA enrichment scores from modules 5, 8, and 9 from lung bronchus premalignant lesion study in our oral PML series. P-values are obtained by ANOVA test.


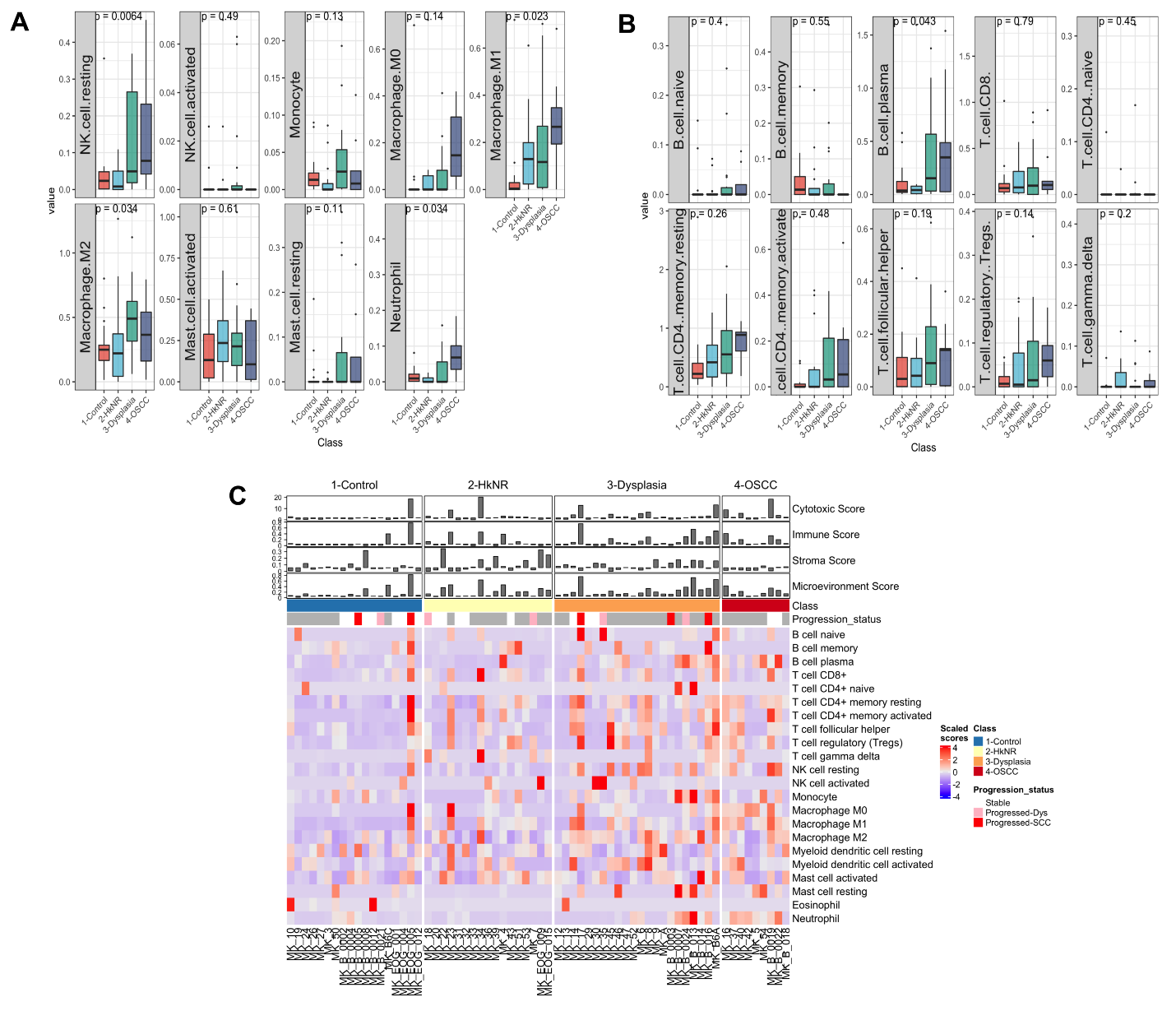


**Figure S4: Cell-type deconvolution scores of immune sub-types.** **A**. innate, **B**. adaptive types. P-values are obtained by ANOVA test. **C**. heatmap of abundances stratified by histopathology along with smoking and progression statuses.


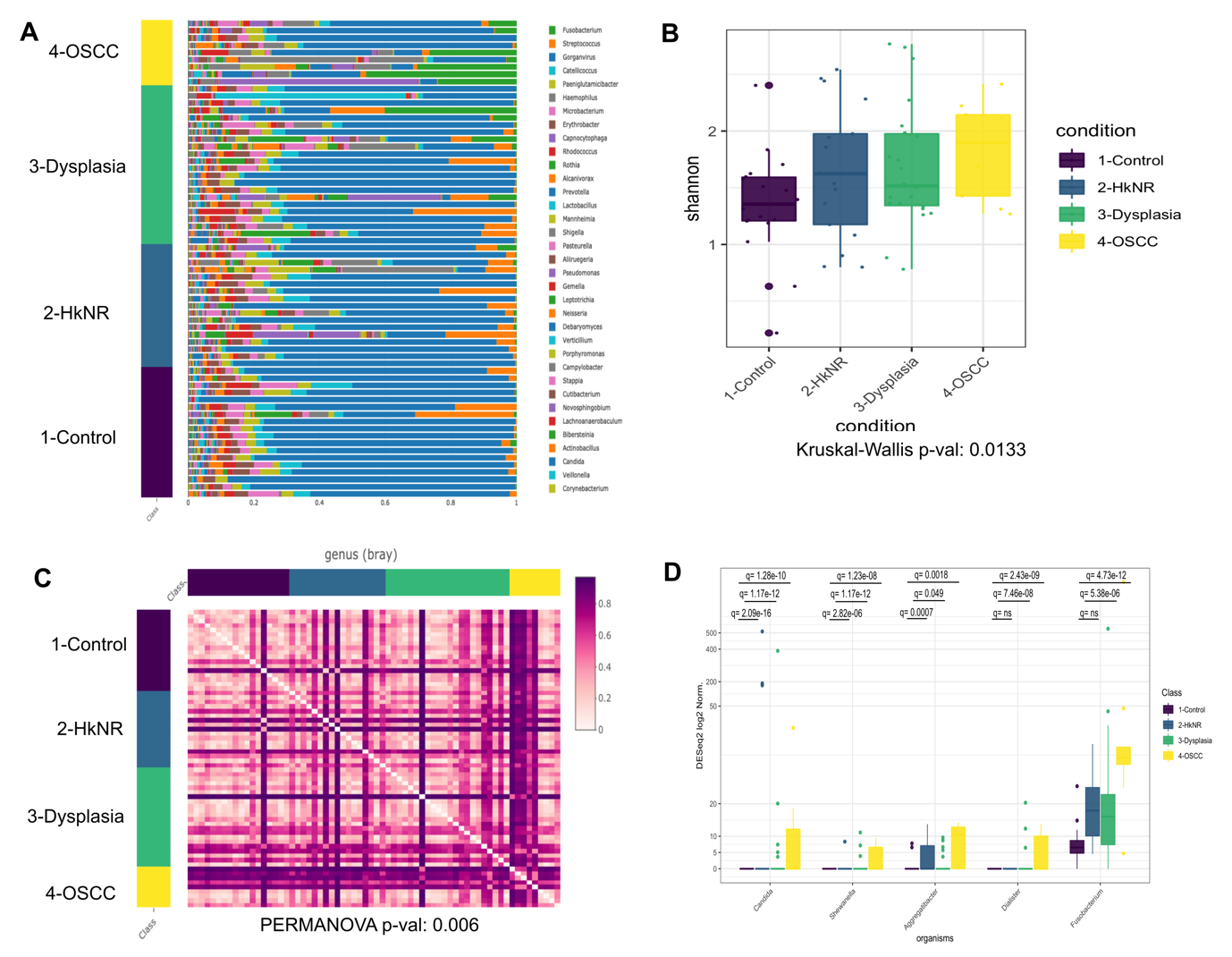


**Figure S5: Microbial diversity analysis**. **A.** Relative abundance of microbial genera across groups with the genus of Fusobacterium in green and Streptococcus in orange. **B**. Alpha diversity stratified by histopathological groups, p-value obtained by Kruskal-Wallis test. **C**. Beta diversity stratified by histopathological groups, p-value obtained by PERMANOVA test. **D**. Relative abundance in DESeq2-normalized and log2-transformed counts of top three differentially abundant species in any of the comparisons of HkNR, Dysplasia, and OSCC with control. Q-values obtained by DESeq2-based analysis.

## Supplementary Tables

ST1. Differential expression analysis results from DESeq2 for pairwise comparisons between the histopathological groups.

ST2. Pathway enrichment analysis of differentially expressed genes pairwise using hyper enrichment analysis using Hallmark compendium.

ST3. Pathway enrichment analysis of differentially expressed genes pairwise using hyper enrichment analysis using Reactome compendium.

ST4. Microbial differential abundant analysis results from DESeq2 for pairwise comparisons between the histopathological groups.

ST5. MSEA results of microbe-set associations with genes.

ST6. Hyper enrichment analysis of microbe-set genes from MSEA on enriched pathways from host analysis.
